# Immediate periodontal bone plate changes induced by rapid maxillary expansion in the early mixed dentition: CT findings

**DOI:** 10.1590/2176-9451.19.3.036-043.oar

**Published:** 2014

**Authors:** Daniela Gamba Garib, Maria Helena Ocké Menezes, Omar Gabriel da Silva Filho, Patricia Bittencourt Dutra dos Santos

**Affiliations:** 1 Full professor, School of Dentistry - University of São Paulo/Bauru.(FOB-USP); 2 MSc in Orthodontics, University São Paulo, UNICID; 3 MSc in Orthodontics, São Paulo State University (UNESP); 4 PhD resident in Applied Dental Sciences (FOB-USP)

**Keywords:** Palatal expansion technique, Periodontium, Spiral computed tomography

## Abstract

**Objective:**

This study aimed at evaluating buccal and lingual bone plate changes caused by rapid maxillary expansion (RME) in the mixed dentition by means of computed tomography (CT).

**Methods:**

The sample comprised spiral CT exams taken from 22 mixed dentition patients from 6 to 9 years of age (mean age of 8.1 years) presenting constricted maxillary arch treated with Haas-type expanders. Patients were submitted to spiral CT scan before expansion and after the screw activation period with a 30-day interval between T1 and T2. Multiplanar reconstruction was used to measure buccal and lingual bone plate thickness and buccal bone crest level of maxillary posterior deciduous and permanent teeth. Changes induced by expansion were evaluated using paired t test (p < 0.05).

**Results:**

Thickness of buccal and lingual bone plates of posterior teeth remained unchanged during the expansion period, except for deciduous second molars which showed a slight reduction in bone thickness at the distal region of its buccal aspect. Buccal bone dehiscences were not observed in the supporting teeth after expansion.

**Conclusion:**

RME performed in mixed dentition did not produce immediate undesirable effects on periodontal bone tissues.

## Abstract (pt)

**Objetivo:**

o presente estudo teve como objetivo avaliar alterações das tábuas ósseas vestibulares e linguais decorrentes da expansão rápida da maxila (ERM), em pacientes na dentição mista, por meio de tomografia computadorizada (TC).

**Métodos:**

a amostra foi constituída por exames de TC helicoidal, realizados de 22 pacientes com dentição mista, dos 6 aos 9 anos de idade (média de 8,1 anos), com atresia maxilar, tratados com expansores do tipo Haas. Os pacientes foram submetidos a tomografia computadorizada helicoidal antes da expansão e após o período de ativação de parafuso expansor, com 30 dias de intervalo entre as fases T_1_ e T_2_. A reconstrução multiplanar foi usada para medir a espessura da tábua óssea vestibular e lingual e a altura da crista óssea alveolar dos dentes posteriores decíduos e dos dentes permanentes. As alterações induzidas pela expansão foram avaliadas usando o teste t pareado (p < 0,05).

**Resultados:**

a espessura das tábuas ósseas vestibular e lingual dos dentes posteriores permaneceu inalterada durante o período de expansão, com exceção dos segundos molares decíduos, que mostraram uma ligeira redução da espessura do osso na região distal. Deiscências ósseas vestibulares não foram observadas nos dentes de suporte após a expansão.

**Conclusão:**

a ERM, realizada na dentição mista, não produziu efeitos imediatos indesejáveis sobre os tecidos ósseos periodontais.

## INTRODUCTION

During rapid maxillary expansion, orthopedic effect is produced by midpalatal suture splitting. Additionally, a dental effect characterized by buccal movement of supporting teeth is also produced.^19-22,27,33^ As a result, maxillary posterior teeth are buccally displaced by an association of inclination and translation. The literature evinces that buccal tooth movement is associated with the occurrence of bone dehiscences. Engelking and Zachrisson,^15^ Steiner et al,^28^ Thilander et al^29^ and Wennströn et al^32^ conducted animal investigations and demonstrated that buccal tooth movement with mild forces increases the distance between the cementoenamel junction and the buccal alveolar crest. Wehrbein et al^31^ reached a similar conclusion when conducting a cadaver study. Buccolingual tooth movement seems to occur through the alveolar bone and not along the bone. It leads to bone dehiscences in the short-term^15,28,29,31,32^ and to gingival recession in the long-term.^1-4^

Recent studies conducted with CT have shown apical migration of buccal alveolar crest of posterior teeth after RME is performed in permanent dentition patients.^18,25^ By means of CT methodology, Garib et al^18^ assessed a sample of eight female adolescents before RME and after the removal of the expander following a 3-month retention period. The authors concluded that RME induced bone dehiscences on the buccal aspect of supporting teeth (first premolars and first molars), especially in subjects who initially presented thinner buccal bone plate. Rungcharassaeng et al^25^ found similar results in a sample of thirty consecutive RME patients with a mean age of 13.8 years. Their findings included buccal bone loss in both horizontal and vertical dimensions for all posterior teeth after expansion, and displayed a significant correlation with age, amount of expansion and initial buccal bone thickness. Ballanti et al^8^ observed, at the end of the active expansion phase, a significant decrease of buccal bone plate thickness of permanent maxillary first molars in a sample of 17 prepubertal patients with a mean age of 11.2 years. However, a tendency for partial recovery was found six months after expansion.

Buccal bone changes produced by rapid maxillary expansion in the permanent dentition raise the question about the periodontal effects of RME performed in the early phases of mixed dentition. During deciduous and early mixed dentitions, RME produces greater orthopedic effects^14,23 ^and transfers anchorage to deciduous molars and canines. A classic implant study showed that, in adolescents, skeletal effects corresponded to 35% of expansion, whereas dental effects accounted for 65%.^23^ On the other hand, in young children, the proportion between skeletal and dental effects was 1:1. Baccetti et al^5^ also observed that RME performed before the peak of skeletal maturation produced more skeletal effects than RME performed after the peak. Thus, the periodontal changes related to the orthodontic effect of RME in the early phases of mixed dentition deserve to be differentiated from those observed in late mixed dentition or even in permanent dentition. Therefore, the aim of this study was to investigate, by means of spiral CT, the periodontal bone changes of RME in early mixed dentition using deciduous teeth as anchorage.

## MATERIAL AND METHODS

This study was approved by the School of Dentistry - University of São Paulo/Bauru Institutional Review Board. The sample comprised spiral CT exams taken from 22 orthodontic patients (10 males and 12 females) with mean initial age of 8.1 years (ranging from 6 to 9 years). The exams had been taken for a previous study,^13^ in 2002, before CBCT was introduced in Brazil. In selecting the sample, the following inclusion criteria were applied: patients in early mixed dentition, and the presence of constricted maxillary arch with or without posterior crossbites. In the examined sample, all subjects were in the early transitional phase, as described by Van der Linden,^30^ and either at stage CS1 or CS2 at the time of treatment.^6^ In other words, patients were treated before or at the beginning of the pubertal growth spurt.

**Figure 1:**
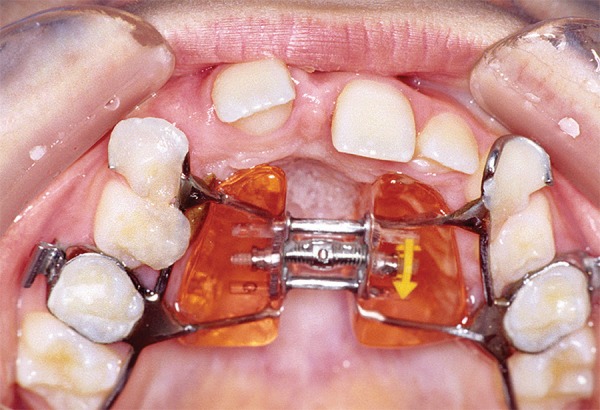
Haas-type expander used in this study.

Maxillary expansion was performed with Haas-type expanders anchored exclusively on deciduous teeth (Fig 1). For all patients, maxillary deciduous second molars functioned as supporting teeth and received bands, whereas maxillary deciduous canines were bonded to a "C" shape anterior extension wire (Fig 1). The expansion screw was activated at two-quarter turns in the morning and two-quarter turns in the evening for approximately 8 days, reaching a mean opening of 7 mm.

All patients were submitted to computed tomography imaging before expansion and after the active expansion phase, with a mean interval of 30 days in between. A spiral computed tomography machine model Xvision EX (Toshiba Corporation Medical Systems Company, Otawara-Shi, Japan) was used at 120 kV and 100 mA, with a scanning time of one second per section. A FC 30 scanning filter, field of view (FOV) of 12.6 x 12.6 cm and matrix of 512 x 512 pixels was used. Window width was 2400 HU with a center of 1300 HU.

For standardization of head positioning in all three planes, the perpendicular light beam resource provided by the machine was used; thereby, allowing comparison of images obtained before and after expansion.^18, 19^ For that purpose, patients were positioned with Camper's plane perpendicular to the ground, while the longitudinal light beam passed through the center of the glabella and the filtrum, and the transverse light beam passed through the lateral eye canthus. Teeth were kept apart in order to avoid imaging the mandibular dental arch. One-millimeter thick axial sections were performed parallel to the palatal plane, including the dentoalveolar and basal areas of the maxilla, up to the lower third of the nasal cavity. The imaged area encompassed 36 to 40 mm, totalizing 36 to 40 sections. This protocol results in images with a spacial resolution that ranges from 0.2 to 0.5 mm.^17^

Data were transferred to a network computer workstation (Silicon Graphics, Toshiba Corporation Medical Systems Company, Otawara-Shi, Japan), using Alatoview software (Toshiba Corporation Medical Systems Company, Otawara-Shi, Japan) on which 2D reformatted images were generated and measured by the computerized method. The Allatoview software generates a very small ball size pointer for linear measurements.

**Figure 2:**
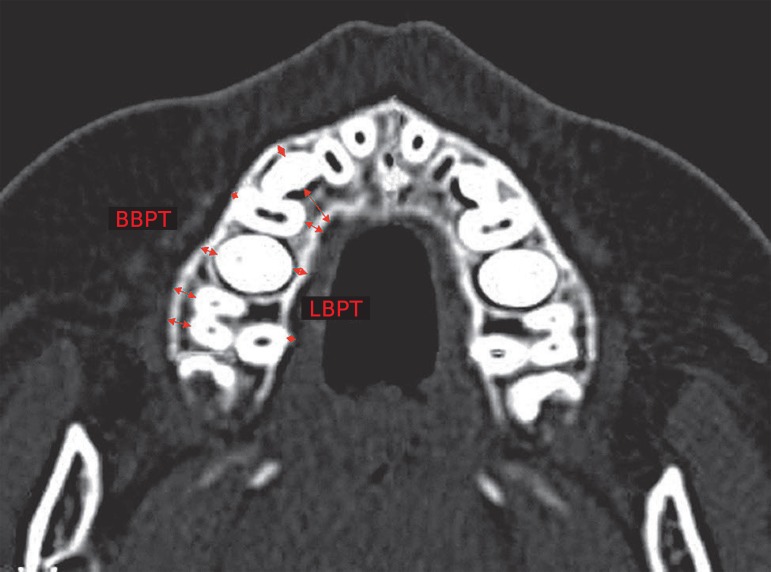
Measurements of buccal and lingual bone plate thickness (BBPT and LBPT) of permanent erupted and non-erupted posterior teeth were performed in the axial section, parallel to the palatal plane, passing at the level of right maxillary permanent first molar furcation.

**Figure 3:**
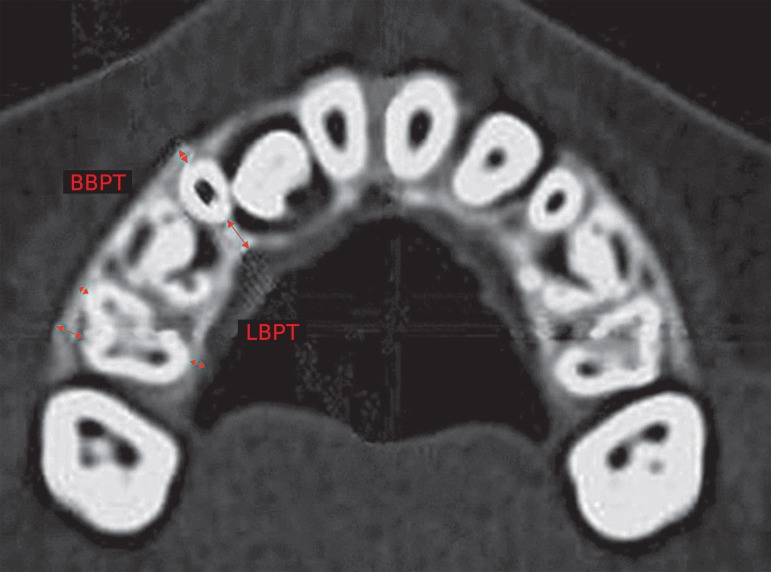
Measurements of buccal and lingual bone plate thickness (BBPT and LBPT) of deciduous posterior teeth were performed in the axial section, parallel to the palatal plane, passing at the level of right maxillary deciduous second molar furcation.

Measurement of alveolar bone plate thickness of maxillary posterior teeth at the buccal and lingual aspects was conducted on two axial sections parallel to the palatal plane, one at the level of right maxillary permanent first molar furcation and another at the level of right maxillary deciduous second molar furcation (Figs 2 and 3). Measurements of alveolar bone thickness were performed on magnified images (4x), before and after expansion. Whenever tooth rotations were present, bone plate thickness was measured at the area where the root was closer to the external contour of the alveolar ridge.

**Figure 4:**
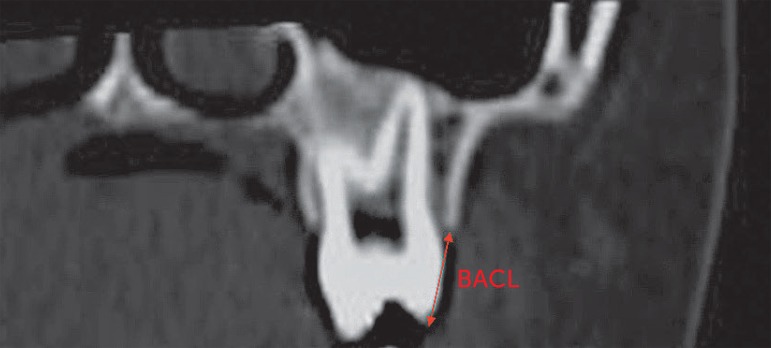
Measurement of maxillary posterior teeth buccal alveolar crest level. BACL: buccal alveolar crest level measured from the buccal cusp tip to the buccal alveolar crest.

Evaluation of the buccal alveolar bone crest level of maxillary posterior teeth was conducted by means of orthoradially reformatted images perpendicular to the contour of the dental arch (cross sections), passing through the center of the buccal aspect of the deciduous canines, deciduous first molar and through the center, mesial and distal areas of the buccal aspect of deciduous second molars and permanent first molars. Figure 4 illustrates the linear variable obtained on each of these eight images both before and after expansion.

### Statistical analyses

All measurements were performed twice within a monthly interval by the same calibrated examiner. Statistical analysis was performed taking into account the mean of these two measurements. Each tooth category corresponded to the mean of right and left side teeth. Mean and standard deviation of each variable were calculated before and after expansion, and so were the changes between these stages. An exploratory test revealed normal distribution of data. Therefore, dependent t-tests were used to compare inter-phase changes. Sample size calculation (with an alfa value of 0.05 and statistical power of 90%) revealed that a sample size of 23 was more than enough. Results were regarded as significant for p < 0.05.

### Systemic and casual errors

Casual and systematic errors were calculated by comparing the first and second measurements with Dahlberg's formula and dependent t-test respectively, at a significance level of 5%.

## RESULTS

The results of the error of the method are shown in Table 1. Two out of 22 variables had a statistically significant systematic error (LBPT of the permanent first molars and BABCL at the center of deciduous second molars - as shown in Figures 2 and 4). Casual errors ranged from 0.11 (BBPT at the distal region of permanent first molars) to 0.52 (BBPT at the mesial region of permanent first molars).

**Table 1:** Systematic and casual errors (dependent t-tests and Dalhberg's formula).

Variables	First	Second	t	p	Dalhberg
	Mean ± SD	Mean ± SD			
**Buccal bone plate thickness (BBPT)**					
Permanent canine germ	1.54 ± 0.60	1.52 ± 0.59	0.260	0.798	0.21
1^st^ premolar germ	0.97 ± 0.46	0.95 ± 0.42	0.356	0.727	0.12
2^nd^ premolar germ	2.10 ± 1.01	2.15 ± 1.17	-0.612	0.549	0.25
Deciduous canine	1.13 ± 0.64	1.15 ± 0.68	0.74	0.463	0.12
Deciduous 2^nd^ molar/mesial	1.11 ± 0.58	1.14 ± 0.59	0.91	0.367	0.12
Deciduous 2^nd^ molar/distal	1.72 ± 0.70	1.75 ± 0.67	0.95	0.349	0.15
Permanent 1^st^ molar/mesial	2.96 ± 1.21	2.77 ± 1.21	1.121	0.277	0.52
Permanent 1^st^ molar/distal	2.92 ± 0.88	2.87 ± 0.90	1.334	0.200	0.11
**Lingual bone plate thickness (LBPT)**					
Permanent canine germ	5.60 ± 2.03	5.72 ± 2.07	-1.170	0.259	0.30
1^st^ premolar germ	3.09 ± 0.97	3.03 ± 1.05	0.637	0.534	0.29
2^nd^ premolar germ	2.71 ± 1.05	2.74 ± 1.02	-0.421	0.680	0.16
Deciduous canine	4.73 ± 1.58	4.79 ± 1.61	1.955	0.057	0.16
Deciduous 2^nd^ molar	1.16 ± 0.55	1.18 ± 0.55	0.64	0.527	0.11
Permanent 1^st^ molar	1.41 ± 0.57	1.30 ± 0.61	3.082	0.007*	0.13
**Buccal alveolar crest level (BACL)**					
Deciduous canine	9.14 ± 2.02	9.28 ± 2.12	-0.690	0.512	0.39
Deciduous 1^st^ molar	7.66 ± 1.16	7.67 ± 1.18	-0.031	0.976	0.27
Deciduous 2^nd^ molar/mesial	7.72 ± 1.40	7.72 ± 1.44	-0.026	0.980	0.35
Deciduous 2^nd^ molar/center	7.80 ± 0.45	7.98 ± 0.40	-2.545	0.020*	0.24
Deciduous 2^nd^ molar/distal	8.10 ± 0.58	8.03 ± 0.62	1.108	0.284	0.18
Permanent 1^st^ molar/mesial	8.39 ± 1.24	8.50 ± 1.08	-1.130	0.275	0.29
Permanent 1^st^ molar/center	7.64 ± 1.02	7.71 ± 1.21	-0.725	0.479	0.27
Permanent 1^st^ molar/distal	7.79 ± 0.88	7.79 ± 0.90	0.000	1.000	0.22

* Statistically significant at P < 0.05

### Buccal and lingual bone plate thickness (BBPT and LBPT)

Table 2 shows that buccal bone plate thickness of supporting teeth was not significantly changed by RME. Additionally, no changes were observed in the buccal bone plate thickness of tooth germs and permanent first molars (Table 2). The only exceptions were the buccal bone plate of first premolar germ which showed a statistically significant decrease (mean of 0.18 mm), and the buccal bone plate of deciduous second molar which showed a statistically significant decrease at the distal region (mean of 0.3 mm).

**Table 2:** Buccal and lingual bone plate thickness expansion changes (Paired t test).

Variables	n	Pre-expansion		Postexpansion		Changes	t	p
		Mean ± SD		Mean ± SD				
**Buccal bone plate thickness (BBPT)**								
Permanent canine germ	23	1.32	0.55	1.31	0.71	-0.01	0.109	0.914
1^st^ Premolar germ	17	0.92	0.41	1.10	0.37	-0.18	-3.318	0.004*
**2nd Premolar Germ**								
Canine	20	2.33	1.08	2.28	1.14	-0.05	0.572	0.574
Deciduous canine	20	1.20	0.82	1.03	0.38	-0.17	0.913	0.373
Deciduous 2^nd^ molar/mesial	19	1.14	0.50	1.06	0.63	-0.09	0.785	0.443
Deciduous 2^nd^ molar/distal	19	1.89	0.69	1.59	0.68	-0.30	2.494	0.023*
Permanent 1^st^ molar/mesial	22	3.21	1.20	2.90	1.32	-0.31	1.478	0.154
Permanent 1^st^ molar/distal	22	3.01	0.75	2.95	0.81	-0.05	0.823	0.420
**Lingual bone plate thickness (LBPT)**								
Permanent canine germ	19	5.81	2.07	5.47	1.98	-0.34	1.627	0.121
1^st^ premolar germ	17	3.46	1.30	3.36	1.15	-0.10	0.580	0.570
2^nd^ premolar germ	18	2.81	1.14	2.85	1.02	0.04	-0.331	0.744
Deciduous canine	21	4.77	1.72	4.81	1.54	0.04	-0.200	0.844
Deciduous 2^nd^ molar	17	1.26	0.53	1.19	0.55	-0.07	0.714	0.486
Permanent 1^st^ molar	20	1.34	0.59	1.42	0.66	0.08	-1.128	0.273

* Statistically significant at P < 0.05

No changes were observed for the lingual bone plate thickness of deciduous and permanent teeth (Table 2).

### Buccal alveolar bone crest level (BABCL)

No statistically significant changes were observed in the level of buccal alveolar bone crest of supporting teeth (deciduous canines and deciduous second molars) and permanent first molars (Table 3).

**Table 3:** Buccal alveolar crest level (BACL) expansion changes (Paired t test).

Variables	n	Pre-expansion	Postexpansion	Change	t	p
		Mean ± SD	Mean ± SD			
Deciduous canine	17	9.02 ± 2.67	8.01 ± 3.55	-1.01	0.934	0.364
Deciduous 1^st^ molar	20	7.32 ± 2.06	7.31 ± 2.07	-0.01	0.088	0.931
Deciduous 2^nd^ molar/mesial	18	7.82 ± 0.67	7.60 ± 1.19	-0.22	0.863	0.400
Deciduous 2^nd^ molar/center	22	7.73 ± 0.57	8.03 ± 0.77	0.30	-1.810	0.085
Deciduous 2^nd^ molar/distal	20	7.82 ± 1.25	8.30 ± 0.93	0.48	-1.714	0.103
Permanent 1^st^ molar/mesial	20	8.31 ± 1.19	8.27 ± 0.80	-0.04	0.190	0.851
Permanent 1^st^ molar/center	21	7.68 ± 0.96	7.59 ± 1.10	-0.09	0.474	0.640
Permanent 1^st^ molar/distal	19	7.91 ± 0.92	7.81 ± 0.84	-0.10	0.495	0.627

*Statistically significant at P < 0.05

## DISCUSSION

Spiral CT proves valuable in assessing alveolar bone thickness and level . Fuhrmann et al^16^ demonstrated that buccal and lingual bone plates can be identified in spiral CT images provided that they are at least 0.5 mm thick. On the other hand, when the periodontal ligament space is apparent, CT identifies even thinner buccal and lingual bone plates (0.2 mm). Measurements taken with spiral CT showed high accuracy and precision.^10,11 ^While periapical radiographs underestimate horizontal alveolar bone defects in 0.6 to 2.2 mm, spiral CT overestimate them in 0.2 mm.^17^ Moreover, previous studies measuring alveolar bone plate thickness and level in spiral CT showed high reproducibility and no-significant errors.^8,18,24^

This study assessed the changes in alveolar bone thickness and level at the region of maxillary posterior teeth after RME was performed during early mixed dentition. The comparison between initial and post expansion CT images was possible due to standardization of head positioning during the exam associated with selection of standardized image sections for measurements. Molar furcation was used as reference to obtain standardized axial sections before and after expansion. This region is relatively stable, since - in reference to the palatal plane - posterior tooth extrusion is very small during RME and may be compensated by tooth buccal tipping.^9^

With regard to changes in buccal bone plate thickness of supporting teeth, deciduous canines, which received a bonded C wire extension, did not reveal reduction in BBPT after expansion (Table 2). Conversely, deciduous second molars, which received bands, showed statistically significant reduction in buccal bone plate thickness at the distal region after expansion, while the mesial aspect of buccal bone plate remained stable (Table 2). The mean decrease in thickness of the distal aspect of buccal bone plate of deciduous second molars was 0.3 mm. Although statistically significant, this reduction was of lesser magnitude than the reduction in BBPT observed for permanent first molars when RME is performed in permanent dentition.^18,25^

RME performed in mixed dentition and anchored exclusively in deciduous teeth produces permanent first molar expansion of one-half the amount of screw expansion, following the orthopedic effect of basal bones.^12^ In this study, palatal wire extension was used at permanent first molars and probably produced further orthodontic effect in this region. However, results revealed that buccal bone plate of permanent first molars did not undergo any changes (Table 2). Previous studies assessing changes in buccal bone plate thickness of posterior teeth after rapid maxillary expansion were performed in permanent dentition^18,25^ or in late mixed and permanent dentitions.^8^ In these previous studies, only permanent teeth were analyzed.^8^ Garib et al^18^ reported a significant decrease in buccal bone plate thickness of banded supporting teeth (first premolars and permanent first molars) that ranged from 0.6 to 0.9 mm, three months after expansion. Rungcharassaeng et al^25^ corroborated the findings by Garib et al^18^ and showed a significant decrease in buccal bone plate thickness of first premolars, second premolars and permanent first molars, with an average of 1.1 mm, 0.8 mm and 1.2 mm, respectively, three months after expansion. Ballanti et al^8^ assessed a sample of young patients aged between 8 to 14 years old, and reported a significant decrease in buccal bone plate thickness of supporting maxillary permanent first molars immediately after the active phase of RME. The mean decrease was less than 0.5 mm and tended to recover six months after expansion.

The effects of RME on mixed dentition are similar to the effects observed in permanent dentition, including an orthopedic effect represented by midpalatal suture split and an orthodontic effect represented by buccal movement of posterior teeth.^13,14^ The V shaped maxillary split observed after RME, in both occlusal and frontal planes during permanent dentition, is also observed during mixed dentition.^13,14^ However, maxillary halves separation is greater in mixed dentition and corresponds to 50% of screw activation, while in permanent dentition it corresponds to approximately 30% of screw activation.^23^ Consequently, the amount of orthodontic effect decreases in mixed dentition in comparison to permanent dentition. Considering that periodontal bone changes are related to tooth movement in the alveolar ridge, it would be expected that RME during the mixed dentition causes less changes in buccal bone plate thickness, as confirmed in this study.

Lingual bone plate thickness (LBPT) of supporting teeth and permanent first molars did not change after RME (Table 2). A previous study performed in permanent dentition reported an increase in lingual bone thickness of posterior teeth after RME, thereby reflecting the buccal movement of these teeth.^18^ This increase had a mean value of 0.7 to 1.4 mm, three months after expansion.^18^ The absence of changes in lingual bone plate thickness in mixed dentition observed in this study can be explained by the smaller amount of orthodontic effects caused by RME during childhood.^5,23^ Furtheremore, the relatively short interval between the first and second CT exam might have influenced the results. A previous study assessing patients in permanent and late mixed dentitions reported that lingual bone plate thickness of permanent first molars did not immediately change after the active phase of expansion; however, an increase was observed 6 months after RME.^8^

All patients comprising the sample had maxillary permanent canines and premolars unerupted when RME was performed. Thus, one of the goals of the present study was to observe the behavior of posterior tooth germs during expansion. Results showed that buccolingual position of posterior tooth germs is not affected by RME. Thickness of buccal and lingual bone plates of posterior tooth germs remained unchanged from T1 to T2 (Table 2). Only the buccal bone plate of first premolars germs demonstrated a small decrease. Such decrease might be related to tooth eruption, although the mean decrease of 0.18 mm is not clinically relevant. It is interesting to note that a favorable side effect of RME in early mixed dentition consists in facilitating eruption of palatally displaced maxillary permanent canines.^7^ This aspect can be regarded as a "bonus" of early expansion therapy at the maxillary arch.

The level of buccal alveolar crest of posterior teeth showed a slight reduction in the distal region of deciduous second molars, only; although changes were not statistically significant (Table 3). Results indicated that bone dehiscences did not occur immediately after RME in the early mixed dentition. In permanent dentition, Garib et al^18^ found that banded teeth showed a significant reduction in buccal alveolar crest level, with a mean loss of 7 mm in the first premolar and 3.5 mm at the mesial aspect of first molars. Rungcharassaeng et al^25^ observed significant vertical bone losses at the buccal aspect of all supporting teeth after RME in permanent dentition. The mean change in buccal crest level of first premolars, second premolars and first molars was 4.4, 1.3 and 2.9 mm, respectively.^25^ Therefore, RME performed in early mixed dentition seems to preserve the integrity of buccal bone plate more than RME performed in permanent dentition. The possible explanation is that the reduced orthodontic effect of RME on mixed dentition in comparison to permanent dentition^5,23^ is not enough for moving posterior teeth throughout the alveolar bone.

Evidence provided by current investigation suggests that early mixed dentition proves adequate to accomplish orthopedic expansion. In addition to good stability^26^ and greater orthopedic effect,^5,23^ RME performed in early mixed dentition may avoid collateral buccal bone changes that predispose gingival recession in the long-term.

## CONCLUSION

RME performed in early mixed dentition did not produce immediate undesirable effects on alveolar bone morphology of maxillary posterior teeth, mainly in terms of bone dehiscences and decrease in buccal bone plate thickness.
